# Predictors of the response to etanercept in patients with juvenile idiopathic arthritis without systemic manifestations within 12 months: results of an open-label, prospective study conducted at the National Scientific and Practical Center of Children's Health, Russia

**DOI:** 10.1186/s12969-017-0178-9

**Published:** 2017-06-14

**Authors:** Ekaterina I. Alexeeva, Leyla S. Namazova-Baranova, Tatyana M. Bzarova, Saniya I. Valieva, Rina V. Denisova, Tatyana V. Sleptsova, Kseniya B. Isaeva, Alexandra M. Chomahidze, Nikolay I. Taibulatov, Anna N. Fetisova, Anna V. Karaseva, Alexandr A. Baranov

**Affiliations:** 1 Federal State Autonomous Institution “National Scientific and Practical Center of Children’s Health” Of the Ministry of Health of the Russian Federation, Moscow, Russia; 2 Federal State Autonomous Educational Institution of Higher Education I.M. Sechenov First Moscow State Medical University of the Ministry of Health of the Russian Federation, Moscow, Russia; 3 Rheumatology Department, Federal State Autonomous Institution “National Scientific and Practical Center of Children’s Health” Of the Ministry of Health of the Russian Federation, Moscow, Russia

**Keywords:** Juvenile idiopathic arthritis, Anti-TNF treatment, Etanercept, Factors associated with treatment response

## Abstract

**Background:**

The aim of this study was to investigate the efficacy of etanercept treatment and to identify predictors of response to therapy within 12 months in patients with juvenile idiopathic arthritis (JIA) without systemic manifestations.

**Methods:**

A total of 197 juvenile patients were enrolled in this study. Response to therapy was assessed using the ACRPedi 30/50/70/90 criteria, the Wallace criteria, and the Juvenile Arthritis Disease Activity Score 71 (JADAS-71). Univariate and multivariate logistic regression analyses were performed to identify potential baseline factors associated with treatment response in different JIA categories.

**Results:**

One year after treatment initiation, 179 (90.9%) patients achieved ACRPedi30; 177 (89.8%) patients achieved ACRPedi50; 168 (85.3%) patients achieved ACRPedi70; and 135 (68.5%) patients achieved ACRPedi90 response. A total of 132 (67.0%) and 92 (46.7%) patients achieved inactive disease according to the Wallace criteria and the JADAS-71 cut-off point, respectively. Excellent response (achieving ACRPedi90 and clinically inactive disease according both to the Wallace criteria and the JADAS71 cut-off point) was associated with persistent oligoarticular JIA category, shorter disease duration before the start of etanercept, a lower number of DMARDs used before the introduction of etanercept, a lower number of joints with limited motion, and lower C-reactive protein at baseline. Poor response (failure to achieve ACR 70 or active disease according to both the Wallace criteria and JADAS71 even when ACR 70 was achieved) was associated with the polyarticular or enthesitis-related JIA categories, higher disease duration before the start of etanercept, and older age at disease onset.

**Conclusion:**

Almost half (45.7%) of the patients who initiated etanercept treatment achieved an excellent response (inactive disease and ACRPedi90) after 1 year. What may be novel is our finding that the response to etanercept therapy was strongly associated with the JIA category. The response to etanercept therapy was also associated with the disease duration before the start of etanercept treatment.

## Background

Juvenile idiopathic arthritis (JIA) is among the most common autoimmune diseases of the musculoskeletal system in children [1, 2]. Its prevalence in Russia is 53.8 per 100,000 children [3]. For each of the 7 JIA categories, there are appropriate recommendations for initial therapy and guidelines for switching medications if they fail or cause serious adverse effects [4].

Searches for novel therapies and response predictors have been initiated because many patients exhibit disease progression and need subsequent treatment when receiving conventional treatments, including MTX [5].

The efficacy of etanercept, a TNFα inhibitor, in daily practice for JIA was confirmed in controlled clinical trials where at least 70% of patients in all categories except RF-positive polyarthritis achieved ACR Pedi 30 and at least 40% of patients in all categories achieved ACR70 [6–8]; CHAQ scores decreased in approximately 53% of patients after 12 weeks of treatment [9], and 41.8% of patients receiving long-term therapy with etanercept (approximately 2.5 years) achieved inactive disease according to the Wallace criteria [10]. Despite the growing use of biological agents that have made it possible for patients to achieve the ACR70 and ACR90 criteria, minimal disease activity and clinical remission still have not been achieved during etanercept treatment in a substantial proportion of cases [6].

Several observational studies with sample sizes ranging from 61 to 863 patients have been published with the aim of determining the factors associated with children’s responses to etanercept treatment [6, 11, 12]. Based on the results of these studies, researchers have identified some factors potentially associated with a better response to etanercept treatment in various combinations: nonsystemic disease onset [13], younger age at disease onset [12, 13], shorter disease duration [6, 13], age at therapy initiation, lower disability scores at therapy initiation (CHAQ and VAS by Physician) [6, 13], higher erythrocyte sedimentation rate (ESR) [13], absence of wrist involvement [12], history of acute anterior uveitis [10], absence of concomitant steroid use [11, 13], and a smaller number of previously used DMARDs [6]. However, no reliable predictors of a good nor poor response to treatment with most drugs have been found because of a lack of consistency in data.

Most of the demographic, clinical, and laboratory characteristics of patients are also associated with the JIA category, defined according to the International League of Associations for Rheumatology (ILAR) classification, that was initially proposed to gather further information on the patterns of clinical presentation [14]. Both certain individual parameters and the comprehensive clinical presentation should be taken into account to create and clarify a reliable model for predicting disease progression and response to etanercept therapy. Further research and analysis of a large body of data are needed to identify the JIA category and clinical characteristics of an “ideal patient” for each modern drug.

Agreement among the conclusions derived from various local data is one of the main criteria for data reliability. There is a clear lack of studies conducted in Russia in the scientific literature. We initiated a prospective study at the Department of Rheumatology of the Scientific Centre for Children’s Health (Moscow) in December 2009 to investigate the efficacy and safety of etanercept treatment, identify the predictors of response to therapy and define the optimal time and conditions for the initiation of etanercept therapy in patients with JIA without systemic manifestations.

## Methods

### Study design and patient enrolment

This open-label, prospective study was conducted at the Department of Rheumatology of the Scientific Centre for Children’s Health, Russian Academy of Medical Sciences, Russia. The children enrolled in the study had taken etanercept between December 2009 and August 2014 and met the following eligibility criteria: ILAR criteria for JIA, no systemic symptoms, no signs of tuberculosis, and being naïve to etanercept before treatment. The study was conducted in compliance with the good clinical practice guidelines to ensure that the data design, implementation, and communication were reliable, that patients’ rights were protected and that the integrity of subjects was maintained through maintaining the confidentiality of their data.

The study was approved by the local Ethics Committee of the Scientific Centre for Children’s Health (protocol no. 36, dated October 16, 2008). All patients and their parents provided written informed consent in accordance with the principles of the Declaration of Helsinki, which included their consent for their data to be used in analyses and to be presented.

### Treatment protocol and data collection

Etanercept was administered via subcutaneous injection at a dose of 0.4 mg per kg body weight (maximum single dose, 25 mg) twice a week. The following parameters were measured and collected at each follow-up point: the JIA disease activity score (JADAS71), physician global assessment of disease activity (0- to 100-mm Visual Analogue Scale, with 0 as the best score, phyVAS); the Childhood Health Assessment Questionnaire (CHAQ; range 0 − 3, with 0 being the best score) for patients or parents, including global assessment of well-being using the Visual Analogue Scale (patVAS); the number of active joints, swollen joints, painful joints, and joints with limited range of motion; erythrocyte sedimentation rate (ESR); C-reactive protein (CRP); and duration of morning stiffness in minutes.

### Assessment of inactive disease

Response to therapy was assessed using the ACR Pedi criteria [7]. The ACR Pedi 30, 50, 70, and 90 response is defined as at least 30%, 50%, 70%, or 90% improvement in 3 or more variables of the JIA core set compared to the baseline, with no more than one variable worsening by more than 30%. We used the JADAS-71 cut-off point [15] and the modified Wallace criteria [16] (no active arthritis, no systemic manifestations, no uveitis, normal ESR (<20 mm/h), duration of morning stiffness ≤15 min, and the physician’s global assessment of disease activity score indicating no disease activity (0 − 10 cm)).

We defined an excellent treatment response as inactive disease after 12 months (the Wallace criteria), achieving the JADAS71 cut-off point, and achieving the ACR 90. A poor treatment response was defined as a failure to achieve ACR 70 or inactive disease according to at least one of the Wallace criteria or the JADAS71 cut-off point, even if ACR 70 was achieved. The intermediate treatment response group comprised all patients not included in the excellent and poor treatment response groups.

### Factors associated with treatment response according to JIA categories

The following potential baseline predictors of response to etanercept treatment were selected based on the literature data: demographic indicators (sex, age at disease onset, disease duration before initiation of etanercept therapy), indicators of disease activity (the number of affected joints; the CHAQ, phyVAS, and patVAS scores; duration of morning stiffness), previous therapy (amount of DMARDs and/or biologicals used), background therapy (oral glucocorticoids, NSAIDs, and sulfasalazine), and laboratory tests (ESR, CRP). Potential predictors were analysed for each of four ILAR categories (persistent oligoarthritis, extended oligoarthritis, rheumatoid factor-negative polyarthritis, and enthesitis-related arthritis).

### Statistical analysis

The R Statistical Package (http://www.r-project.org) was used for calculations. Descriptive statistics were reported as absolute frequencies or as median values with IQR. Depending on the type of the processed data, we used either the Mann-Whitney U test, the Pearson’s chi-squared test, or Fisher’s exact test and the non-parametric Kruskal-Wallis analysis of variance by rank and median for multiple comparisons.

Univariate and multivariate logistic regression analyses were used to determine the significance of potential predictors of the response to etanercept treatment among baseline indicators (comparing excellent response to poor and intermediate response combined and comparing poor response to intermediate and excellent responses combined). Independent parameters for modelling were chosen based on statistical and clinical significance and correlations. The results are presented as adjusted odds ratios (OR; the OR for each covariate was adjusted to the effects of the other covariates) with 95% confidence intervals; *P* values were calculated.

All the reported *P* values were based on two-tailed significance tests; *P* values <0.05 were considered statistically significant. We used STATISTICA 7.0 software (StatSoft, Tulsa, USA) and RStudio software version 0.99.484 (Free Software Foundation, Inc., Boston, USA) with R packages version 3.2.2 (The R Foundation for Statistical Computing, Vienna, Austria) for the analyses.

## Results

### Baseline characteristics of the complete cohort

The study initially included 198 children with JIA who had begun etanercept therapy; 137 (69.2%) were females [Table 1]. One male patient withdrew early from the study because of a severe allergic reaction to etanercept. Hence, 197 children were enrolled in the efficacy analysis. Since there was only one patient (1; 0.51%) in each of the polyarticular RF positive and psoriatic arthritis groups, univariate and multivariate efficacy analyses could not be performed for these categories.

**Table 1:** Characteristics of children with JIA starting treatment with etanercept (*N* = 197) at baseline

Characteristics	All patients (*n* = 197)	Persistent oligoarticular	Extended oligoarticular	Polyarticular RF negative	Enthesitis-related
		(*n* = 84)	(*n* = 23)	(*n* = 64)	(*n* = 24)
Female, n (%)	137 (69.5)	68 (81.0)	16 (69.6)	46 (71.9)	5(20.8)^d^
Age at JIA onset, median (IQR), years	3 (2 – 7)	2.3 (1.7 – 3.7)^a^	2 (1.5 – 4)	3.25 (2 – 7.5)^a^	9 (5 – 11.8)^d^
Age at start of etanercept, median (IQR), years	7.25 (4 – 12)	5.1 (2.9 – 8.9)^a^	7.5 (3.5 – 10)	9 (5.8 – 13)^a^	13 (10.9 – 15.0)^d^
Disease duration before start etanercept, median (IQR), years	2.1 (1 – 5)	1.9 (0.9 – 4)^a^	3 (1.7 – 7)	3.5 (1.8 – 6)^a^	3 (1.5 – 5)
History of chronic anterior uveitis, n (%)	7 (3.6)	3 (3.6)	1 (4.3)	3 (4.7)	0 (0)
History of at least 1 DMARD use except MTX, n (%)	84 (42.6)	24 (28.6)^ab^	7 (30.4)^c^	34 (53.1)^a^	18 (75.0)^bc^
History of at least 1 DMARD use, n (%)	170 (86.3)	69 (82.1)	22 (95.7)	57 (89.1)	22 (91.7)
History of ≥2 DMARDs use, n (%)	76 (38.6)	21 (25.0)^ab^	6 (26.1)	32 (50.0)^a^	15 (62.5)^b^
History of at least 1 biologic use, n (%)	44 (22.3)	10 (11.9)^a^	5 (21.7)	20 (31.3)^a^	7 (29.2)
History of ≥2 biologics use, n (%)	7 (3.6)	1 (1.2)	0 (0)	5 (7.8)	1 (4.2)
History of oral steroid use, n (%)	55 (27.9)	8 (9.5)^ab^	7 (30.4)	30 (46.9)^a^	8 (33.3)^b^
Concurrent MTX use, n (%)	136 (69.0)	51 (60.7)^a^	21 (91.3)^ab^	51 (79.7)	12 (50.0)^b^
Concurrent sulfasalazin use, n (%)	6 (3.0)	1 (1.2)	0 (0)	2 (3.1)	3 (12.5)
Concurrent oral steroid use, n (%)	10 (5.1)	0 (0)	2 (8.7)	5 (7.8)	3 (12.5)
NSAID use, n (%)	121 (61.4)	44 (52.4)	15 (65.2)	43 (67.2)	18 (75.0)

Paired superscript letters (^a^, ^b^, ^c^) denote the existence of differences between two groups in each line (*p* < 0.05). The superscript letter ^d^ denotes the difference between indicators in one group and those in all other groups. *RF* rheumatoid factor, *IQR* interquartile range, *DMARD* disease-modifying antirheumatic drugs, *MTX* Methotrexate, *NSAID* nonsteroidal anti-inflammatory drugs

At baseline, the median patient age was 7.25 years (IQR 4 − 12 years), and the disease duration was 2.1 years (IQR 1 − 5 years). The median CHAQ score was 1.25 (IQR 0.5 − 1.875).

At baseline, 136 (69.0%) patients were receiving concomitant treatment with MTX; 10 (5.0%) were receiving concomitant treatment with oral steroids; 6 (3.0%) were receiving concomitant treatment with sulfasalazine; and 121 (61.4%) were receiving NSAIDs. No patients received any intra-articular steroids during the entire study period. One hundred sixty-three (82.7%) patients had a history of using at least one DMARD, and 17 (8.6%) patients had previously received two or more DMARDs. Etanercept was the first biological agent taken by 153 (77.7%) patients. Seven (3.6%) patients had previously used two or more biologicals.

### Baseline characteristics according to JIA categories

An analysis of the data across JIA categories revealed a significant difference in the baseline parameters among different JIA types. In the enthesitis-related group, male patients predominated considerably over females (20.8% females). In the other three groups (persistent oligoarticular, extended oligoarticular, and polyarticular RF-negative), female patients were prevalent (81.0%, 69.6%, and 71.9%, respectively). The median age at JIA onset in the enthesitis-related group differed significantly from that of the other groups: 9 years for the enthesitis-related JIA group (min-max 1-16 years) compared with 2.3 years (min-max 10 months – 15 years) in the persistent oligoarticular group, 2 years (min-max 6 months – 12 years) in the extended oligoarticular group, and 3.25 years (min-max 8 months – 13 years) in the polyarticular RF-negative group. The persistent oligoarticular and polyarticular RF-negative groups differed significantly in terms of treatment duration (1.9 vs 3.5 years, respectively). The patient groups differed significantly in terms of previous treatment. Less than one-third of the patients in the oligoarthritis groups had a history of using at least one DMARD other than MTX (28.6 and 30.4% in the persistent oligoarticular and the extended oligoarticular groups, respectively). Meanwhile, the percentage of these patients in the polyarticular RF-negative and enthesitis-related groups was >50% (53.1 and 75%, respectively). The administration of biologicals (11.9%) and oral steroids (9.5%) was minimal in the persistent oligoarticular group.

### Response to therapy within 12 months

Of the 197 patients enrolled in the study, 17 (8.6%) discontinued etanercept within the first year: 5 due to the occurrence of AEs (three patients had acute infusional reactions, and two had drug-induced hepatotoxicity), five due to primary failure, two due to de novo uveitis, and one due to uveitis flare; four additional patients withdrew because of non-adherence to therapy. Of the remaining 180 patients, 36 withdrew during the first year: six discontinued the study due to age (they were transferred to adult care centres), and 30 were transferred to local medical centres for further follow-up.

According to the final measurements taken after 12 months, 179 (90.9%) patients achieved ACR Pedi 30,177(89.8%) patients achieved ACR Pedi 50, 168(85.3%) patients achieved ACR Pedi 70, 135(68.5%) patients achieved ACR Pedi 90; 132(67.0%) patients achieved inactive disease according to the Wallace criteria; and 92 (46.7%) patients achieved inactive disease according to the JADAS cut-off point [Tables 2 and 3].

**Table 2:** Dynamic of basic clinical and laboratory parameters among children with JIA starting treatment with etanercept (*N* = 197) at baseline and at the final measurement during 1 year follow-up

Characteristics	Baseline, median (IQR)	1 year follow-up, median (IQR)
Active joint count	4 (2-10)	0 (0-3)
Persistent oligoarticular	2 (2-4)	0 (0-0)
Extended oligoarticular	6 (5-10)	0 (0-0)
Polyarticular RF-	14 (6-23.5)	0 (0-2)
Enthesitis-related	5 (2.5-9)	0 (0-1.5)
Limited joint count	4 (2-11)	0 (0-2)
Persistent oligoarticular	2 (2-3.5)	0 (0-0)
Extended oligoarticular	6 (3-10)	0 (0-1)
Polyarticular RF-	15 (6.5-25.5)	0 (0-6)
Enthesitis-related	4.5 (4-9)	0 (0-1.5)
Swollen joint count	4 (2-8)	0 (0-0)
Persistent oligoarticular	2 (2-4)	0 (0-0)
Extended oligoarticular	6 (4-6)	0 (0-0)
Polyarticular RF-	9 (5-12.5)	0 (0-2)
Enthesitis-related	4 (2-7)	0 (0-0)
Pained joint count	4 (2-10)	0 (0-0)
Persistent oligoarticular	2 (1.5-3)	0 (0-0)
Extended oligoarticular	6 (3-9)	0 (0-0)
Polyarticular RF-	12 (4-23)	0 (0-0)
Enthesitis-related	4.5 (3-9)	0 (0-1)
Morning stiffness, min	30 (0-60)	0 (0-15)
Persistent oligoarticular	30 (0-60)	0 (0-0)
Extended oligoarticular	35 (0-60)	0 (0-3)
Polyarticular RF-	60 (20-120)	0 (0-0)
Enthesitis-related	60 (15-120)	0 (0-1)
Physician global of disease (0-100 mm)	60 (45-80)	5 (0-15)
Persistent oligoarticular	50 (42-70)	0 (0-4)
Extended oligoarticular	65 (46-80)	3 (0-14)
Polyarticular RF-	70 (56-86)	6 (2-19)
Enthesitis-related	68.5 (54.5-82.5)	4.5 (0.5-11)
Parent/patient global of well-being (0-100 mm)	68 (50-82.5)	6.5 (2.8-14.3)
Persistent oligoarticular	60 (49-70)	2 (0-7)
Extended oligoarticular	68 (50-81)	5 (3-10)
Polyarticular RF-	73.5 (60-87)	9 (2-15.5)
Enthesitis-related	70 (50-90)	4.5 (0-12)
Childhood health assessment questionnaire (0-3)	1.3 (0.5-1.9)	0 (0-0.2)
Persistent oligoarticular	0.78 (0.5-1.5)	0 (0-0)
Extended oligoarticular	1.5 (1-1.9)	0 (0-0.25)
Polyarticular RF-	1.75 (13-2.25)	0 (0-0.75)
Enthesitis-related	1.5 (0.75-2.5)	0 (0-0.25)
Juvenile arthritis disease activity score-71	19.2 (13.8-28.5)	1.1 (0.3-3.9)
Persistent oligoarticular	14.9 (11.7-18.9)	0.5 (0-1.1)
Extended oligoarticular	21.8 (16.3-28.5)	1 (0-2.5)
Polyarticular RF-	29.6 (20.6-39.8)	1.45 (0.65-6.4)
Enthesitis-related	20.6 (15.6-30.7)	1.1 (0.1-3.6)
Erythrocyte sedimentation rate, mm/h	21 (12-35)	5 (3-11)
Persistent oligoarticular	19.5 (10-28)	5 (2-7)
Extended oligoarticular	20 (15-35)	4 (2-12)
Polyarticular RF-	25.5 (16-41.5)	5 (3-15.5)
Enthesitis-related	30 (8.5-59.5)	5 (4-13.5)
C-reactive protein, mg/l	6.6 (1.9-19.7)	1 (0-4.6)
Persistent oligoarticular	2.6 (1-10)	0 (0-1)
Extended oligoarticular	6.4 (2.4-15)	1 (0-3)
Polyarticular RF-	9.8 (4-27)	1 (0-6.5)
Enthesitis-related	16.7 (7.6-52.3)	3.7 (0.9-12.6)

*RF* rheumatoid factor, *IQR* interquartile range

**Table 3:** Final disease activity parameters achieved in different ILAR categories during one year etanercept treatment

JIA subtype	ACR Pedi, %				Inactive disease, %
	ACR Pedi 30	ACR Pedi 50	ACR Pedi 70	ACR Pedi 90	
All patients (*n* = 197)	90,9	89,8	85,3	68,5	67
Persistent oligoarticular (*n* = 84)	90,5	90,5	88,1	77,4	86,9
Extended oligoarticular (*n* = 23)	95,7	95,7	95,7	73,9	65,2
Polyarticular RF negative (*n* = 64)	90,6	87,5	85,4	57,8	45,3
Polyarticular RF positive (*n* = 1)	100	100	0	0	0
Psoriatic (*n* = 1)	100	100	100	100	100
Enthesitis-related (*n* = 24)	87,5	87,5	83,3	62,5	58,3

*n* number, *RF* rheumatoid factor, *JADAS-71* juvenile arthritis disease activity score 71; inactive disease defined by Wallace criteria

Of 197 patients, 90 (45.7%) achieved an excellent response to etanercept therapy after 1 year, while 48 (24.4%) were considered poor responders. The remaining 59 (29.9%) were considered intermediate responders.

**Fig. 1:**
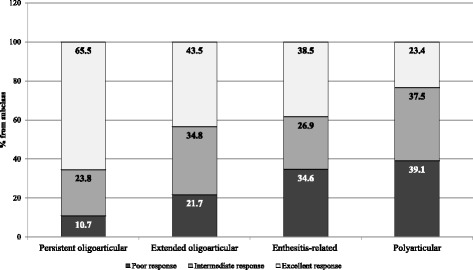
Distribution of responses to etanercept in different JIA categories within 12 months after beginning treatment

We found a significant relationship between diagnosis according to JIA category and the response level. In the patients with persistent oligoarticular JIA, the percentage of excellent responders (65.5%) after 1 year of etanercept treatment was significantly higher than that in polyarticular RF-negative patients (23.4%) and in enthesitis-related arthritis patients (37.5%) [Fig. 1]. More than-one third of the patients in the polyarticular RF-negative and enthesitis-related groups were poor responders (39.1% and 33.3%, respectively). The highest percentage of patients who achieved inactive disease was observed in the group with persistent oligoarticular JIA (86.9%), whereas the lowest percentage was observed among the polyarticular RF-negative patients (45.3%).

All 7 patients with a history of uveitis before starting etanercept treatment had no active uveitis at the time of etanercept initiation. Uveitis flare occurred in one patient (0.5%) during the first year of treatment, resulting in the discontinuation of etanercept. The patient was subsequently switched to abatacept. Two cases of de novo uveitis (1%) developed 6 months after the initiation of etanercept therapy among all patients without a history of uveitis: one patient started etanercept monotherapy at the age of 10.7 years, and the second patient started receiving etanercept in combination with MTX at the age of 3.9 years. After the discontinuation of etanercept, both patients were switched to adalimumab.

### Analysis of predictors of response to etanercept within 12 months

For each JIA category, we analysed the factors that initially differed in the patients with different response levels. Univariate analysis of all baseline characteristics was performed for each diagnosis. For the multivariate analysis in each group of patients, we selected independent factors associated with response to therapy based on the univariate analysis data and literature data. We used logistic regression to determine the significance of the variables in the univariate and multivariate analyses [Tables 4 and 5].

**Table 4:** Univariable and multivariable analysis of factors associated with response to etanercept for polyarticular RF-negative and enthesitis-related JIA

Baseline characteristics	Poor vs Intermediate and Excellent Responders				Excellent vs Intermediate and Poor Responders			
	Univariable analysis		Multivariable analysis		Univariable analysis		Multivariable analysis	
	Odds ratio (95% CI)	*p* value	Odds ratio (95% CI)	*p* value	Odds ratio (95% CI)	*p* value	Odds ratio (95% CI)	*p* value
Polyarticular RF-negative JIA								
Demographic								
Gender	0.886 (0.519-1.461)	0.642			1.334 (0.765-2.283)	0.295		
Disease duration before start of etanercept	0.55 (0.312-0.917)	0.027	*0.313 (0.116-0.730)*	*0.012*	0.995 (0.573-1.818)	0.985		
Age of disease onset	0.659 (0.391-1.087)	0.106	*0.418 (0.176-0.866)*	*0.028*	1.535 (0.84-3.14)	0.193	*0.518 (0.267-0.945)*	*0.039*
Disease activity								
CHAQ score at start of etanercept	0.58 (0.321-0.984)	0.053	0.801 (0.300-2.108)	0.649	1.745 (0.982-3.255)	0.065	1.089 (0.469-2.543)	0.841
VAS disease activity by patient/parent at start of etanercept	0.946 (0.565-1.562)	0.828			1.1 (0.617-1.934)	0.741		
VAS disease activity by physician at start of etanercept	0.76 (0.436-1.266)	0.308			1.375 (0.785-2.444)	0.261		
No. of joints with LOM	0.575 (0.328-0.96)	0.041	0.438 (0.150-1.129)	0.103	2.081 (1.069-4.712)	0.049	*0.430 (0.185-0.900)*	*0.033*
Morning stiffness, min	0.505 (0.259-0.876)	0.026	0.524 (0.214-1.130)	0.121	4.592 (1.54-20.62)	0.021	0.680 (0.326-1.312)	0.267
Previous therapy								
No. of DMARDs used before start of etanercept	0.638 (0.366-1.065)	0.095	1.272 (0.558-3.069)	0.573	0.707 (0.386-1.253)	0.243		
Fact of biologics using before start of etanercept	0.696 (0.42-1.146)	0.155	0.604 (0.283-1.224)	0.170	0.891 (0.513-1.589)	0.685		
Laboratory tests								
CRP at start of etanercept	0.712 (0.41-1.171)	0.189	0.658 (0.304-1.333)	0.252	3.361 (1.166-19.326)	0.088	*0.539 (0.271-0.959)*	*0.046*
Enthesitis-related JIA								
Demographic								
Gender	-	-			0.6 (0.242-1.365)	0.226		
Disease duration before start of etanercept	0.17 (0.021-0.59)	0.029	*3.980 (1.294-23.095)*	*0.048*	3.219 (0.995-19.183)	0.12	0.385 (0.051-1.531)	0.263
Age of disease onset	2.286 (0.885-7.397)	0.114			0.404 (0.13-1.002)	0.073	1.200 (0.308-5.023	0.789
Previous therapy								
No. of DMARDs used before start of etanercept	0.737 (0.275-1.816)	0.51			3.587 (1.261-15.132)	0.039	0.351 (0.047-1.416)	0.205
Fact of biologics using before start of etanercept	0.419 (0.161-0.997)	0.056	2.030 (0.714-6.369)	0.185	2.052 (0.81-8.276)	0.184	1.046 (0.220-4.252)	0.948

All entries in italic are statistically significant

**Table 5:** Univariable and multivariable analysis of factors associated with response to etanercept for extended and persistant oligoarticular JIA

Baseline characteristics	Poor vs Intermediate and Excellent Responders				Excellent vs Intermediate and Poor Responders			
	Univariable analysis		Multivariable analysis		Univariable analysis		Multivariable analysis	
	Odds ratio (95% CI)	*p* value	Odds ratio (95% CI)	*p* value	Odds ratio (95% CI)	*p* value	Odds ratio (95% CI)	*p* value
Extended oligoarticular JIA								
Demographic								
Disease duration before start of etanercept	0.449 (0.146-1.144)	0.114	1.066 (0.184-7.261)	0.942	1.729 (0.723-4.87)	0.245	*6.808 (1.520-63.332)*	*0.036*
Age of disease onset	0.561 (0.195-1.363)	0.213			2.091 (0.788-9.563)	0.218	3.447 (1.106-15.630)	0.053
Disease activity								
VAS disease activity by physician at start of etanercept	0.361 (0.1-0.98)	0.07	0.841 (0.173-3.997)	0.816	1.271 (0.543-3.149)	0.582		
Pain joint count	0.838 (0.333-2.186)	0.695			0.668 (0.242-1.577)	0.373	0.340 (0.070-1.097)	0.104
Previous therapy								
No. of DMARDs used before start of etanercept	0.533 (0.19-1.343)	0.198	0.518 (0.087-2.841)	0.431	1.064 (0.449-2.613)	0.885		
Fact of biologics using before start of etanercept	0.497 (0.188-1.193)	0.123	0.440 (0.138-1.337)	0.136	1.794 (0.729-6.57)	0.253		
Persistant oligoarticular JIA								
Demographic								
Disease duration before start of etanercept	0.671 (0.385-1.216)	0.157	1.118 (0.522-2.736)	0.785	1.154 (0.731-1.807)	0.527		
Disease activity								
Morning stiffness, min	0.746 (0.429-1.391)	0.302			1.386 (0.886-2.25)	0.16	1.448 (0.902-2.413)	0.129
Previous therapy								
Oral corticosteroids before start of etanercept	0.681 (0.389-1.095)	0.093	0.927 (0.482-5.718)	0.858	0.768 (0.149-1.297)	0.487		
No. of DMARDs used before start of etanercept	0.633 (0.343-1.171)	0.131	0.562 (0.250-1.232)	0.145	1.629 (1.025-2.748)	0.049	*1.691 (1.04-2.958)*	*0.046*
Background therapy								
Concomitant NSAID at start of etanercept	0.838 (0.408-1.637)	0.609			0.711 (0.445-1.12)	0.145	0.649 (0.388-1.058)	0.089
Laboratory tests								
CRP at start of etanercept	0.869 (0.515-1.791)	0.635			1.3 (0.833-2.099)	0.247	1.385 (0.874:2.274)	0.164

*CHAQ* child health assessment questionnaire, *CI* confidence interval, *DMARD* disease-modifying antirheumatic drug, *ESR* erythrocyte sedimentation rate, *JIA* juvenile idiopathic arthritis, *VAS* visual analog scale

All entries in italic are statistically significant

For the persistent oligoarthritis group, no factor was significant for poor response according to the multivariate analysis. In the group of excellent responders, a lower number of DMARDs was associated with better response.

In the extended oligoarticular group, none of the factors was significant for poor response according to the multivariate analysis. Excellent response was associated with a shorter disease duration.

In the polyarticular RF-negative group, poor response to treatment was associated with longer disease duration and older age. Excellent response was associated with a smaller number of joints with limited functions, a lower CRP level at the initiation of etanercept therapy, and younger age at disease onset.

In the enthesitis-related group, poor response to therapy was associated with longer disease duration. No significant factors were associated with excellent response.

## Discussion

This open-label study of children with JIA without active systemic manifestations provides evidence supporting the efficacy and safety of the first course of etanercept treatment in the largest cohort of JIA patients in Russia. According to an intention-to-treat analysis after 12 months of etanercept treatment, only 18 (9.1%) patients failed to achieve ACR Pedi 30, while 135 (68.5%) patients achieved ACR Pedi 90 and 132 (67.0%) patients achieved inactive disease according to the Wallace criteria. The JIA categories differed in terms of the level of response to ETA. Hence, to determine the optimal time and conditions for initiating etanercept treatment in JIA patients without systemic manifestations, we identified baseline predictors associated with excellent and poor responses to treatment in the studied JIA categories.

Previous research on the efficacy of anti-TNF drugs, and etanercept in particular, demonstrated different responses to treatment in JIA patients with systemic manifestations [17]. However, differences in other diagnoses have not been examined in many studies. In one study [18], the authors attempted to determine the contribution of diagnosis to the response to treatment but did not find any significant differences. This may be explained by the relatively small sample size (24 patients in two oligoarticular categories and 13 patients in the polyarticular category). We enrolled only patients without systemic manifestations because the Scientific Centre for Children’s Health uses different treatment strategies for these categories of patients, as published previously [19]. Since the literature indicates that many demographic and clinical characteristics (onset; further characteristics of the arthritis; disease course; the presence of ANA, chronic or acute anterior uveitis, HLA allelic associations, etc.) are actually determined by the JIA category [14], we decided to analyse the response individually for each category. Because we found that the patients within the diagnostic groups differed in their response to therapy, we analysed the predictors for each JIA category.

We used the clustering method for the response to therapy to single out three levels (poor, intermediate, and excellent response) according to the results of 1 year of etanercept treatment. Other researchers have focused on analyses using a similar approach [6, 11, 12] since it is promising for building predictive models and elaborating the algorithms for treatment selection. The results of the analysis enabled us to identify the consistency and inconsistency of our results with real-world data on the efficacy of etanercept treatment.

The results of various studies show that some baseline demographic characteristics can predict treatment response. The factors identified among the literature data are as follows: female sex [6], age at initiation of treatment [7, 13], age of JIA onset [6, 12], and disease duration [6, 12, 20]. We found that sex was not a significant predictor of response, regardless of diagnosis. However, because polyarthritis and oligoarthritis are characterized by female predominance, while male predominance is typical of only enthesitis-related arthritis, the associations between sex and response level that some researchers have found may be due to the relationship between sex and diagnosis.

In our study, the cohorts of patients in different JIA categories had different age characteristics. Shorter disease duration was associated with excellent response in the extended oligoarthritis group, while longer disease duration was associated with poor response in the polyarthritis and enthesitis arthritis groups. The higher efficacy of etanercept in patients in the early disease stage (up to 1.4 − 2 years of disease) may indicate a “window of opportunity” when the use of etanercept appears to be more effective if there are no irreversible changes in the joints.

The patient’s past medical history is very important when prescribing treatment for JIA. Studies have separately assessed the effect of previous use of different classes of medications [6]. In our study, only the number of previously used DMARDs was a significant predictor of response in the persistent oligoarthritis group.

Among all the laboratory parameters analysed in our study, only lower CRP was a significant predictor of excellent response in polyarticular patients.

Indicators of joint involvement are very important characteristics of the clinical presentation in JIA patients. In our study, the small number of joints with limitation of motion in children with arthritis correlated with an excellent response to etanercept treatment. This is related to the fact that a large number of joints with LOM are observed in children who experienced the onset of arthritis at an older age (median, 6 years) and who have been suffering for a long time and therefore experienced irreversible changes in joints and tendons.

At the beginning of the study, only 7 patients had a history of JIA-associated uveitis, but none of them had active uveitis when etanercept therapy was initiated. A history of this disease was insignificant in the multivariate analysis because of the small sample size. However, it should be mentioned that etanercept was discontinued within the first year of treatment in 2 of these7 patients: one because of uveitis flare (the patient administered a combination of etanercept and methotrexate) and the other because of treatment nonadherence. In addition, two children without a history of uveitis developed it after 6 months of etanercept use. Hence, the total rate of uveitis or uveitis flares was 3 cases (1.5%) within the first year of treatment: one case using monotherapy and two cases using combination therapy with methotrexate. This rate is somewhat lower than the data reported by other authors, particularly the large cohort of children from the Italian Registry) [10, 21], which could be due to both population features and differences in the total cohort of patients with respect to age and JIA category. Nevertheless, meticulous long-term monitoring of children receiving etanercept therapy is needed to reveal the development of risks and associations.

In our study, the VAS scores for disease activity as assessed by physician and patients were insignificant in the univariate analysis regardless of diagnostic category. The CHAQ score was significant in the univariate analysis for the polyarticular RF-negative group in the poor response model but was insignificant in the multivariate analysis.

However, the current study had some limitations. Despite its prospective design, the study lacked a control group. Hence, we could not assess the identified predictors as prognostic markers and could not evaluate whether a different treatment would be more effective than etanercept therapy for the poor responders. We did not consider immunological and genetic parameters as predictors of treatment response. Researchers are currently extensively investigating these parameters, so their value as predictors of treatment response may be determined in the future.

## Conclusion

Our findings demonstrated that the response to etanercept therapy was strongly associated with the JIA category. While our results confirm earlier findings that etanercept is an effective and safe medication that resulted in ACR 90 and inactive disease in 45% of children with JIA after 1 year of treatment, few clinical, laboratory, and historical parameters can predict treatment success. The revealed predictors of treatment efficacy included persistent oligoarticular JIA, a shorter disease duration before the initiation of etanercept therapy, a smaller number of DMARDs used before the initiation of etanercept therapy, and a smaller number of joints with LOM. Lower C-reactive protein levels at baseline were a laboratory predictor. Polyarticular and enthesitis-related arthritis with a longer disease duration before the initiation of etanercept were predictors of poor response to etanercept treatment. These factors may help physicians to identify patients who might benefit from earlier treatment with etanercept.

### Key messages

Almost half of children with JIA achieved an excellent response to etanercept treatment after 1 year.

Children with persistent oligoarthritis and a shorter disease duration were more likely to achieve an excellent response to etanercept treatment.

## Abbreviations

CRP: C-reactive protein
DMARD: Disease-modifying antirheumatic drug
JADAS71: JIA disease activity score
JIA: Juvenile idiopathic arthritis;
MTX: Methotrexate
NSAID: Nonsteroidal anti-inflammatory drug
